# Short-Term Voice Changes After Endotracheal Intubation: Comparative Study of Head/Neck and Abdominal Surgery

**DOI:** 10.3390/jcm15166324

**Published:** 2026-08-16

**Authors:** Ivana Šimić Prgomet, Renata Curić Radivojević, Goran Augustin, Drago Prgomet

**Affiliations:** 1 Department of Otolaryngology, Head and Neck Surgery, University Hospital Centre Zagreb, 10000 Zagreb, Croatia; ivana.simic.prgomet@kbc-zagreb.hr (I.Š.P.); drago.prgomet@kbc-zagreb.hr (D.P.); 2 Faculty of Education and Rehabilitation Sciences, University of Zagreb, 10000 Zagreb, Croatia; 3 Department of Anesthesiology, Reanimatology, Intensive Medicine and Pain Therapy, University Hospital Centre Zagreb, 10000 Zagreb, Croatia; rcuric@kbc-zagreb.hr; 4 School of Medicine, University of Zagreb, 10000 Zagreb, Croatia; 5 Department of Surgery, University Hospital Centre Zagreb, 10000 Zagreb, Croatia

**Keywords:** endotracheal intubation, head and neck surgery, abdominal surgery, voice disorders, acoustic analysis

## Abstract

**Background/Objectives**: Endotracheal intubation can cause postoperative voice disturbances, even after short procedures. These effects have been documented in head and neck surgery, but direct comparisons between head/neck and abdominal surgeries are lacking. We sought to compare short-term postoperative voice changes in patients undergoing head/neck and abdominal surgery and to evaluate their association with intubation parameters and patient factors. **Methods**: We prospectively studied 80 adults (mean age X ± SD) undergoing either head–neck surgery (parotidectomy or total thyroidectomy) or abdominal surgery. Voice acoustic parameters (fundamental frequency (F_0_), jitter, shimmer, intensity, maximum phonation time (MFT)) were measured preoperatively and on postoperative days 2, 14, and 30. Anesthetic parameters were recorded during the perioperative period. Anesthesiologists were blinded to the ongoing investigation. We used repeated-measures ANCOVA (with sex as a covariate) to test for group-by-time interactions and main effects. Effect sizes were calculated. **Results**: Eighty patients were included. All acoustic parameters showed significant postoperative changes (*p* < 0.001). The most pronounced alterations occurred in the early postoperative period, with decreased F_0_, intensity, and MFT, and increased jitter and shimmer. Significant interaction effects between time and type of surgery were observed for F_0_ (*ηp*^2^ = 0.445), shimmer (*ηp*^2^ = 0.210), intensity (*ηp*^2^ = 0.267), and MFT *(ηp*^2^ = 0.149), indicating distinct recovery patterns across groups. Patients undergoing abdominal surgery showed less pronounced and more rapidly resolving changes, whereas head and neck surgery was associated with greater and more persistent impairment. Among all parameters, shimmer demonstrated the largest overall effect size (*ηp*^2^ = 0.338), suggesting high sensitivity to short-term voice changes. Perioperative factors, including duration of surgery, endotracheal tube size, and BMI, were significantly associated with the extent of voice changes. **Conclusions**: Short-duration endotracheal intubation can lead to measurable short-term voice changes, which are more pronounced after head/neck surgery (particularly total thyroidectomy). Objective acoustic analysis detected these subclinical alterations even when patients were asymptomatic. These findings underscore the importance of monitoring voice outcomes and optimizing intubation techniques, although future work is needed to correlate acoustic changes with laryngeal exam findings.

## 1. Introduction

Endotracheal intubation is a standard procedure used to maintain airway patency during surgical procedures. Although generally safe, it may result in postoperative laryngeal complications, including voice, swallowing, and breathing disorders. Hoarseness and vocal fatigue are among the most commonly reported symptoms, even after short-duration intubation, particularly in head and neck surgery [1,2]. Thyroid surgery has been associated with postoperative voice disorders even in the absence of laryngeal nerve injury, while parotid gland surgery (PGS) may also affect voice due to anatomical proximity and surgical manipulation [1,3,4,5,6]. In contrast, abdominal surgeries are not directly related to laryngeal structures and may serve as a useful comparison group [7]. Despite this, research comparing the short-term effects of endotracheal intubation on voice quality across surgical specialties remains limited. Furthermore, it remains unclear to what extent postoperative voice changes are influenced by intubation itself, the type and duration of surgery, or patient-related factors [8]. To our knowledge, few studies have systematically evaluated short-term changes in objective acoustic voice parameters following endotracheal intubation across different surgical populations [9,10,11,12,13]. This study aims to address this gap by comparing voice outcomes in head and neck and abdominal surgery.

Parotidectomy and total thyroidectomy were grouped together as “head and neck surgery” because both share the surgical field’s proximity to the larynx, comparable positioning and shared-airway conditions during intubation, and comparable postoperative soft-tissue edema in the neck—features that distinguish them from abdominal surgery, which does not involve the neck. This grouping was used only to contrast neck-region procedures against a non-neck control; all group-level comparisons and post hoc analyses in this study nonetheless treat PGS and TT as distinct groups, allowing differences between these two procedures to be examined directly rather than obscured by pooling.

## 2. Materials and Methods

This prospective, comparative, observational study was designed to examine differences in the intensity and duration of voice disorders after endotracheal intubation among patients undergoing different types of surgery (Parotidectomy (PGS), Total Thyroidectomy (TT), and Abdominal Surgery). The study also examined correlations between objective vocal parameters, operation duration, tube size, and the patient’s BMI. A formal sample size calculation was not performed. The sample size was determined by the number of eligible patients during the study period.

### 2.1. Ethics Approval and Patient Consent

The study protocol complies with the ethical standards of the Institutional Review Board at University Hospital Centre Zagreb and with the Declaration of Helsinki. The study was approved by the Institutional Review Board on 25 September 2024 (document class 8.1-23/209-3, no. 02/013 AG). Each patient signed the informed consent to participate in the study.

### 2.2. Participants

Patients scheduled for elective head and neck surgery at the Department of Otolaryngology and Head and Neck Surgery, Phoniatric Reference Center, or elective abdominal surgery at the Outpatient Surgery Unit of the University Hospital Center Zagreb were included in the study after signing informed consent. A formal a priori sample size calculation was not performed because this was a prospective observational study based on consecutive eligible patients treated during the predefined study period. The study was conducted from 1 November 2024 to 1 January 2026. Therefore, the sample size reflected the number of patients meeting the inclusion criteria rather than a predetermined recruitment target. The inclusion criteria were adult age (18–70 years; this upper limit was a typographical error and should read 18–86 years, consistent with the age range reported in Table 1), normal preoperative voice status, and normal laryngeal status as determined by laryngoscopy. No formal upper age limit was applied; eligibility was based on fitness for elective surgery and normal preoperative voice/laryngeal status, which is why one participant in the PGS group was 86 years old. Patients with preoperatively diagnosed pathologic vocal cord findings (nodules, polyps, vocal cord paralysis), postoperative injuries of the vocal cords or the superior and recurrent laryngeal nerves, laryngopharyngeal reflux, and those in whom postoperative voice evaluation could not be performed due to uncooperativeness were excluded from the study.

Patients were divided into three groups based on the type of surgery: 30 underwent PGS, 30 underwent TT, and 20 underwent abdominal surgery. All surgeries were performed by experienced surgeons who performed more than a hundred procedures per year.

### 2.3. Voice Acoustic Analysis Protocol

The Voice Acoustic Analysis Protocol used in this study was previously published by Šimić et al. [8]. All acoustic measurements were performed under standardized conditions to minimize variability related to the recording environment and patient performance. Recordings were scheduled during the mid-morning hours (typically between 09:00 and 12:00) at each visit for a given patient, in a quiet examination room, before any strenuous vocal use that day, to limit potential circadian and vocal-loading effects on the acoustic measures. Data were collected at four time points: (1) preoperatively; (2) the first postoperative measurement (POM1) was performed on the second day after surgery; (3) the second postoperative measurement (POM2) was performed 14 days after surgery; and (4) the third postoperative measurement (POM3) was performed one month after surgery. Three speech samples were collected for each patient at each measurement, and 5 s from the middle of the second phonation signal were analyzed. Acoustic voice analysis included fundamental frequency (F_0_), intensity, jitter, shimmer, and maximum phonation time (MFT). F_0_, also known as voice pitch, is measured in Hertz (Hz). The average voice pitch is 220–225 Hz in women, 100–150 Hz in men, and around 300 Hz in children. Intensity is defined as voice volume; it is measured in decibels (dB), with an average of 60 dB. Jitter indicates perturbations in vocal cord vibration at the fundamental frequency; it is measured as a percentage (%) and is considered normal in the range of 0 to 0.5%. Shimmer indicates intensity-related perturbations in vocal cord vibration; it is measured as a percentage and is considered normal in the range of 0 to 5%. MFT is measured in seconds (s) during habitual phonation.

### 2.4. Endotracheal Intubation

All patients underwent endotracheal intubation for airway management during surgery. The choice of intubation technique (direct or video laryngoscopy) and endotracheal tube size was left to the anesthesiologist in charge. Detailed data on intubation technique, including the use of a stylet or bougie, the number of intubation attempts, cuff pressure, and tube lubrication, were not systematically recorded because anesthesiologists were blinded to the study and data were collected retrospectively. No supraglottic airway devices were used in the included patients. These factors are recognized as potential contributors to laryngeal trauma and represent a limitation of this study.

The investigators who performed and analyzed the acoustic voice recordings were not part of the surgical or anesthesia teams and were not involved in intraoperative decision-making; however, because the surgical group was evident from the patient’s clinical context (e.g., neck incision, hospital ward), formal masking of the acoustic analysts to surgical group assignment was not feasible. Because the acoustic parameters were derived from objective, automated signal analysis rather than subjective auditory-perceptual judgment, the risk of assessment bias from this lack of masking is considered low, though it cannot be entirely excluded and is noted as a limitation.

### 2.5. Statistical Analysis

Data were analyzed using repeated-measures ANCOVA to assess differences across time points and between groups undergoing different types of surgery. Time was treated as the within-subject factor, surgery group as the between-subject factor, and sex as a covariate. The assumption of sphericity was assessed with Mauchly’s test, and Greenhouse–Geisser corrected results were reported when violated. Post hoc pairwise comparisons were performed using Bonferroni adjustment when appropriate. Effect sizes were expressed as partial eta squared (*ηp*^2^). Pearson correlation was used. Partial eta squared values were interpreted using conventional benchmarks (~0.01 small, ~0.06 medium, ~0.14 large effect). Data analysis was performed in SPSS 28 [14]. Sex was included as a covariate because it was unevenly distributed across the study groups and is a well-established determinant of normal acoustic voice characteristics. Age and BMI were not included as covariates because they did not differ significantly between the study groups. Although surgery duration differed between groups (Table 1), it was not included as a covariate in the ANCOVA model; its association with voice outcomes was instead examined separately using Pearson correlations (Tables 4–6). Because surgery duration and tube size are interrelated with surgery type, this approach cannot fully separate their independent contributions from that of surgery type, which is acknowledged as a limitation.

## 3. Results

**Figure 1:**
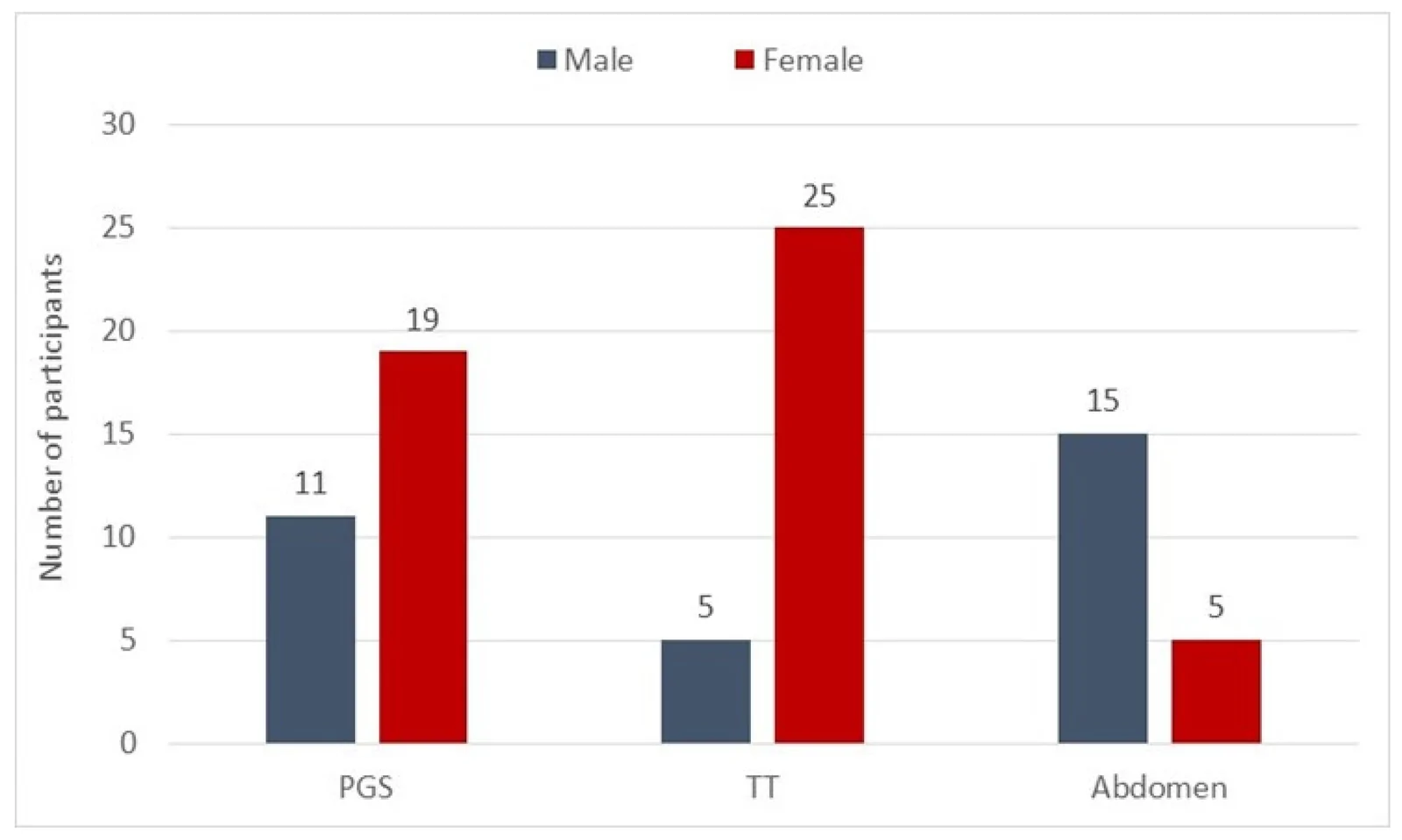
Sex differences (absolute numbers) in patients per group. PGS—parotidectomy, TT—total thyroidectomy, Abdomen—abdominal surgery.

Thirty-one male and forty-nine female patients were enrolled. Significantly more men were in the abdominal surgery group (χ^2^ = 17.292, *p* = 0.00) than in the head–neck groups (Figure 1). All 80 enrolled patients completed the full assessment protocol, with no dropouts and no missing acoustic measurements at POM1, POM2, or POM3 (N = 80 at each time point, Table 2). Thus, attrition bias did not affect the results.

Descriptive statistics for tube size, participants’ BMI, surgery duration, and age are presented in Table 1. Significant differences in tube size and surgery duration were observed across operation groups, whereas participants were evenly distributed across groups by age and BMI. The difference in average tube size between groups was attributable to sex (since there is no other reason for using different tube sizes across different types of surgery). Therefore, it was appropriate to retain sex as a covariate in further analyses.

Initially, we calculated descriptive statistics, including skewness and kurtosis, for voice parameters at all time points to assess the distributions of the results (Table 2). Although the Kolmogorov–Smirnov test is often used to assess the distribution of results, some authors believe it is overly sensitive to small deviations from normality and can yield false-positive results [15,16]. Given that repeated-measures ANOVA is robust, an overview of skewness and kurtosis as indicators of the distribution of results is sufficient.

All observed objective voice parameters were within the normal range preoperatively, indicating that patients did not have voice disorders before their surgery. However, at POM1, the mean values across all observed parameters changed, indicating the development of a voice disorder. Over time, the observed parameters gradually returned to normal; however, even one month after surgery, some remained outside the normal range (Table 2).

Because mean values can mask interindividual variability, we estimated, from the distribution of POM3 values in Table 2, the approximate proportion of patients still outside the accepted normal range: for jitter (normal 0–0.5%; POM3 M = 0.53%, SD = 0.424), approximately half of patients (~53%) remained above the upper normal limit, and for shimmer (normal 0–5%; POM3 M = 6.59%, SD = 3.936), approximately two-thirds of patients (~66%) remained above the upper normal limit at one month. These figures are distribution-based approximations rather than exact patient-level counts, but they indicate that a clinically meaningful proportion of patients had not fully normalized by POM3, reinforcing that group means alone underestimate the persistence of subclinical voice change.

Table 3 shows the main effects of time and type of surgery, and their interaction, on observed voice parameters.

**Figure 2:**
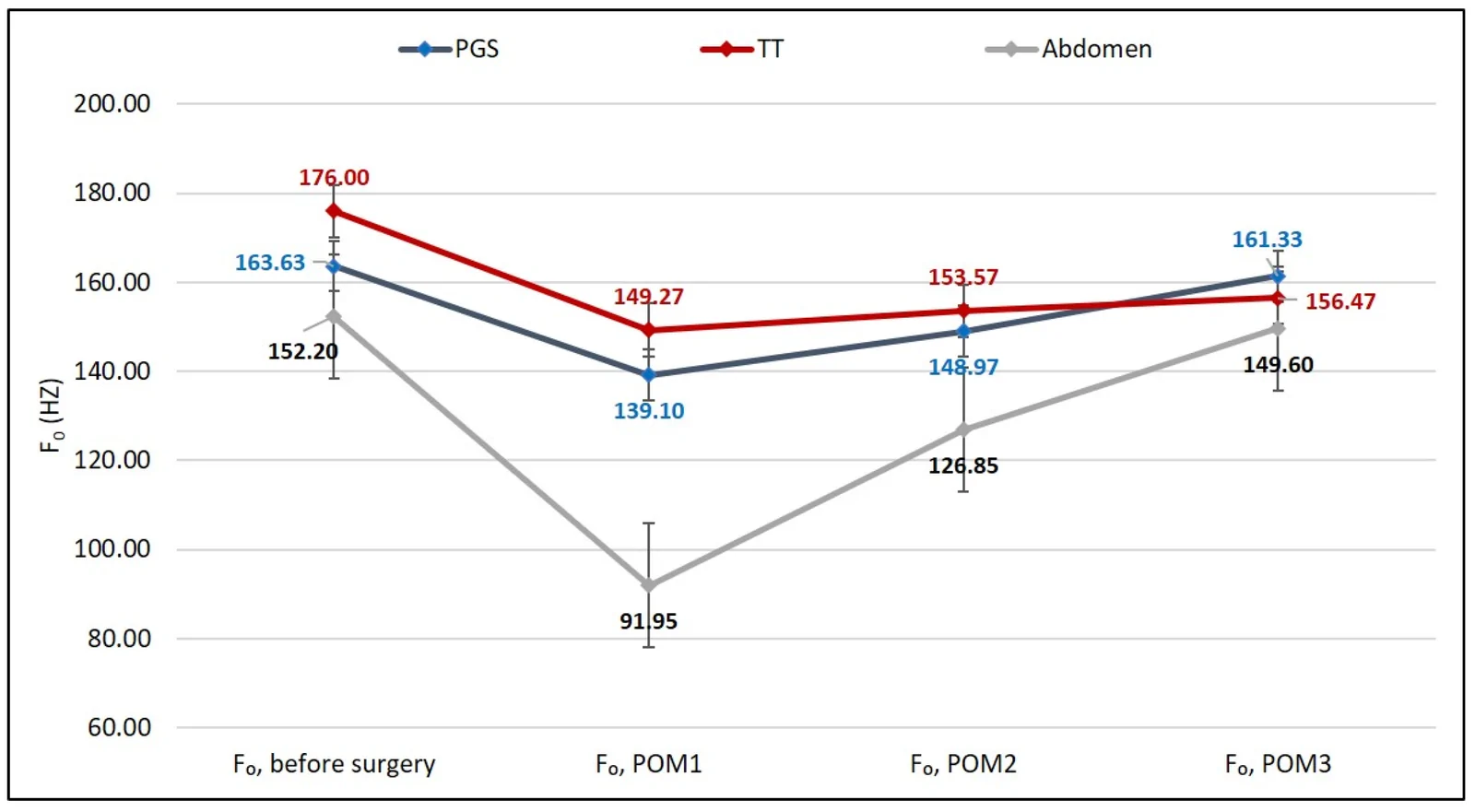
Interaction effect of time and surgery type on F_0_. POM—postoperative measurement, F_0_—fundamental frequency (voice pitch), PGS—parotid gland surgery, TT—total thyroidectomy, Abdomen—abdominal surgery.

**Figure 3:**
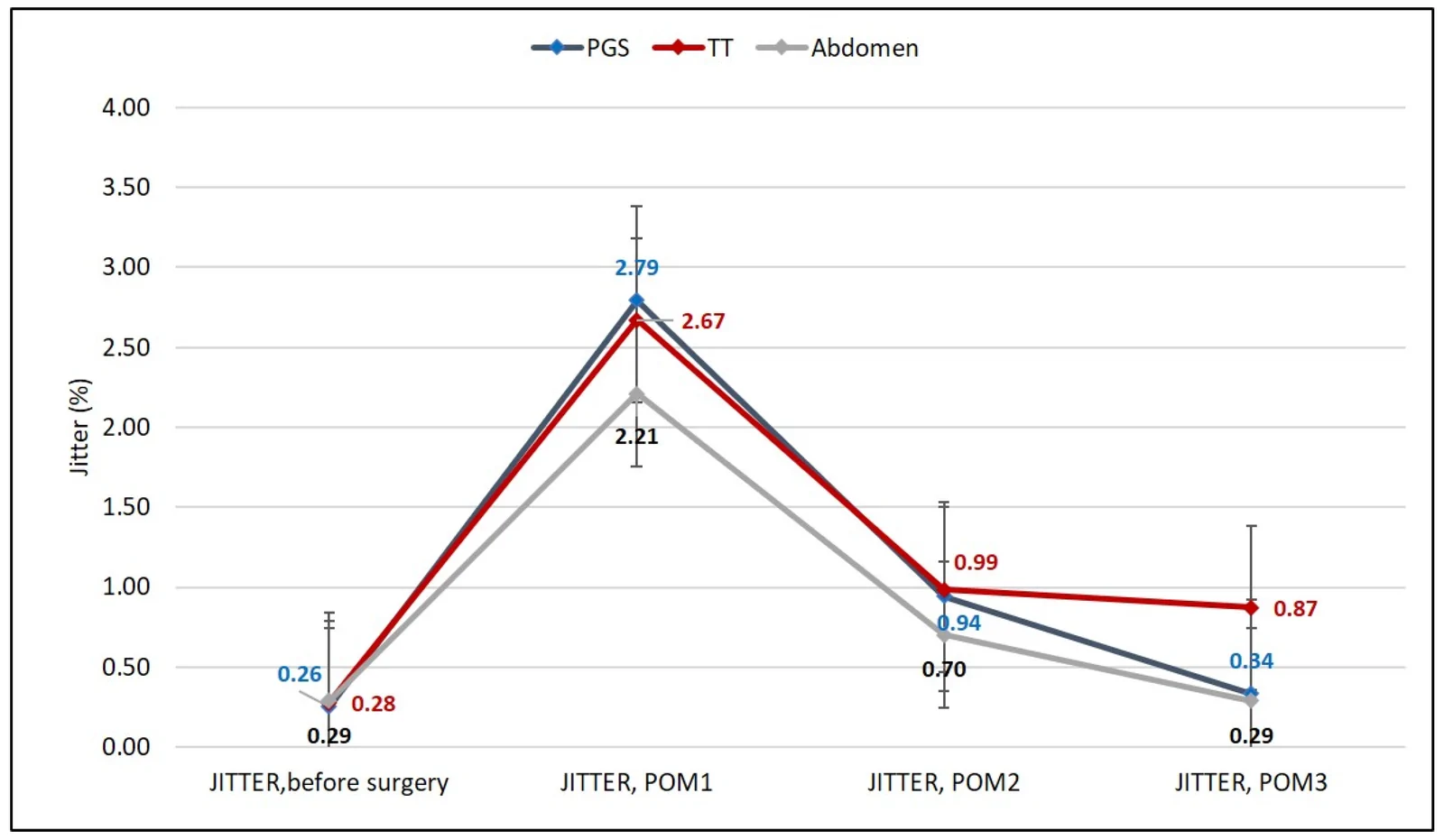
Interaction effect of time and surgery type on Jitter. POM—postoperative measurement, PGS—parotid gland surgery, TT—total thyroidectomy, Abdomen—abdominal surgery.

**Figure 4:**
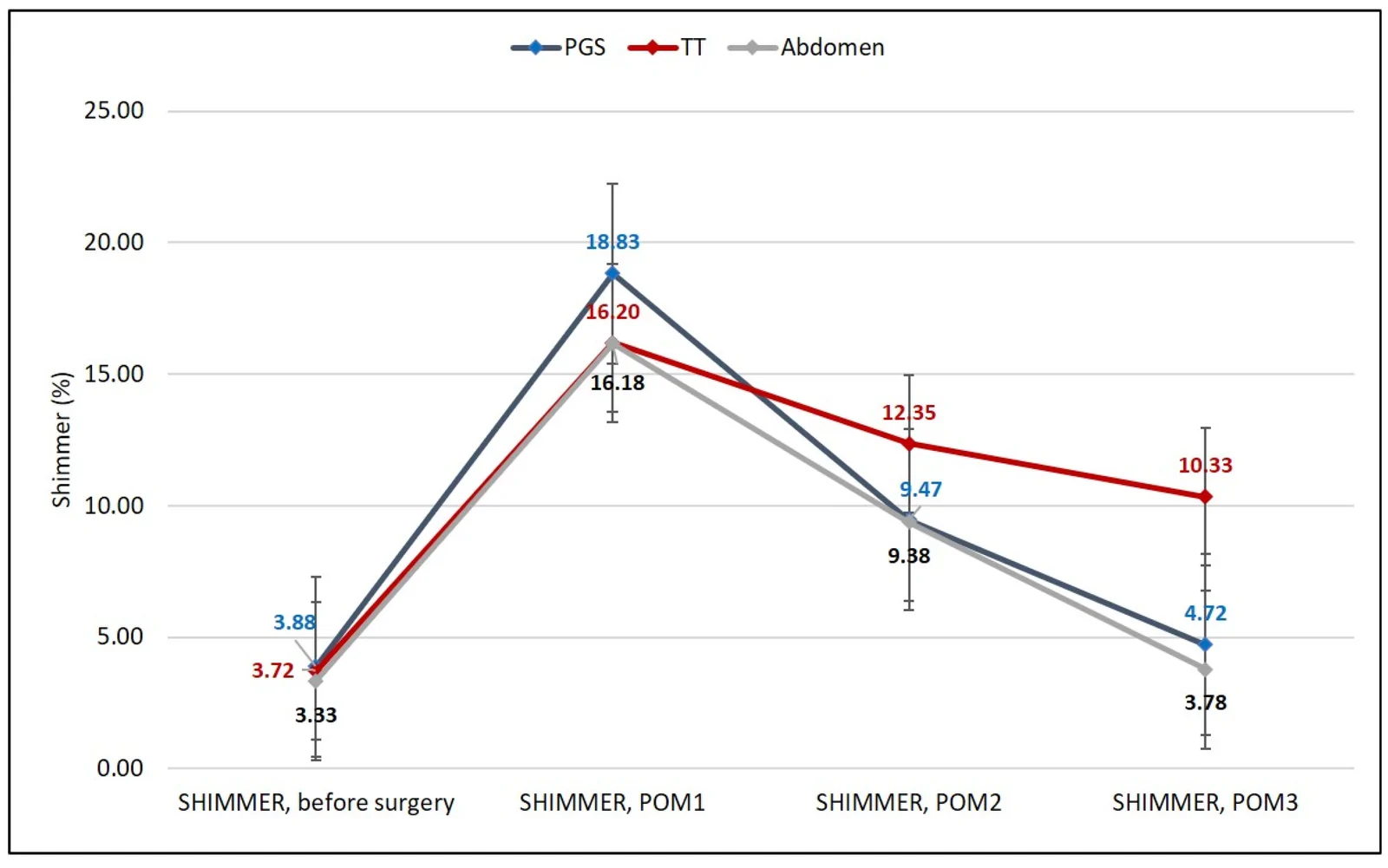
Interaction effect of time and surgery type on Shimmer. POM—postoperative measurement, PGS—parotid gland surgery, TT—total thyroidectomy, Abdomen—abdominal surgery.

**Figure 5:**
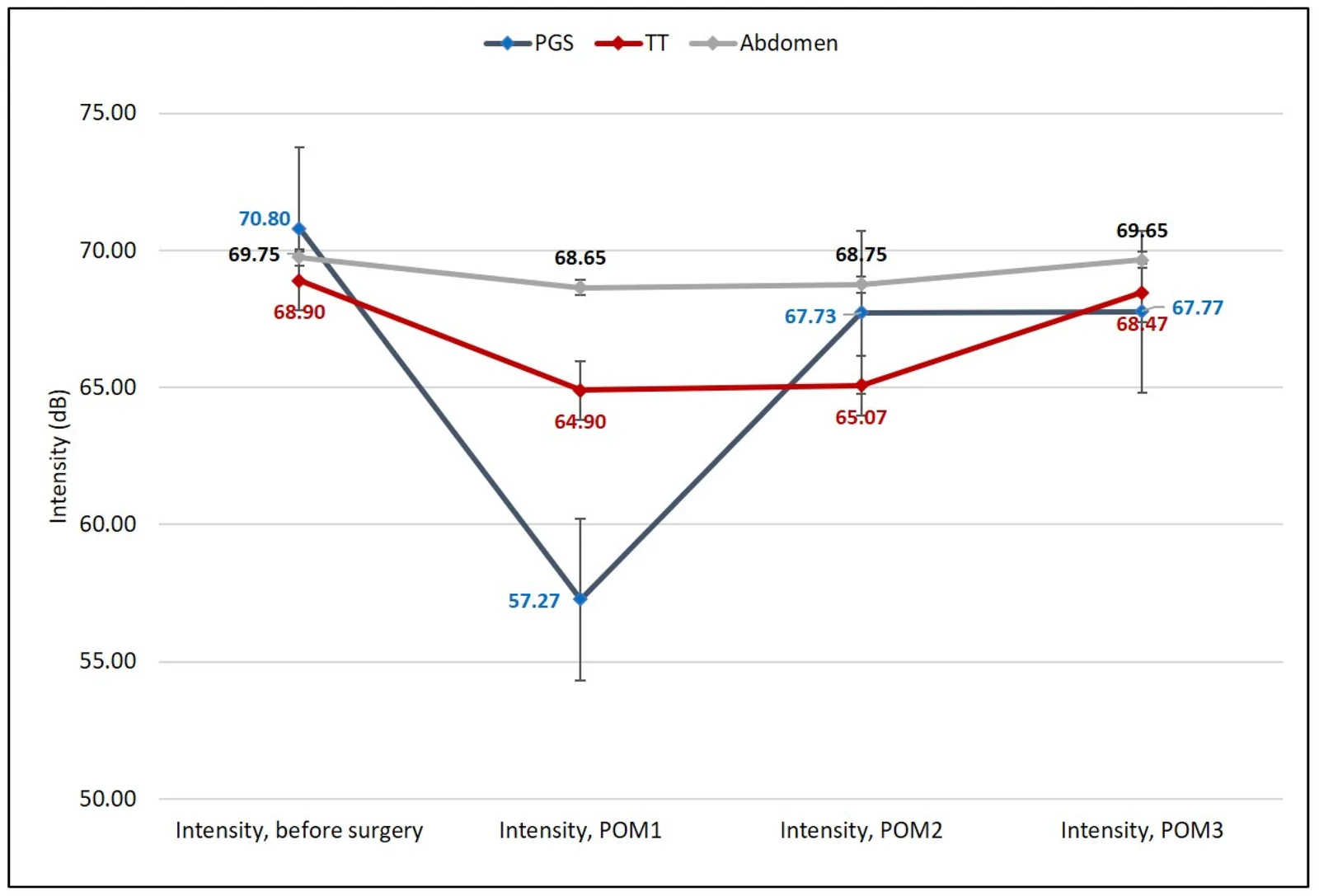
Interaction effect of time and surgery type on Intensity. POM—postoperative measurement, PGS—parotidectomy, TT—total thyroidectomy, Abdomen—abdominal surgery.

**Figure 6:**
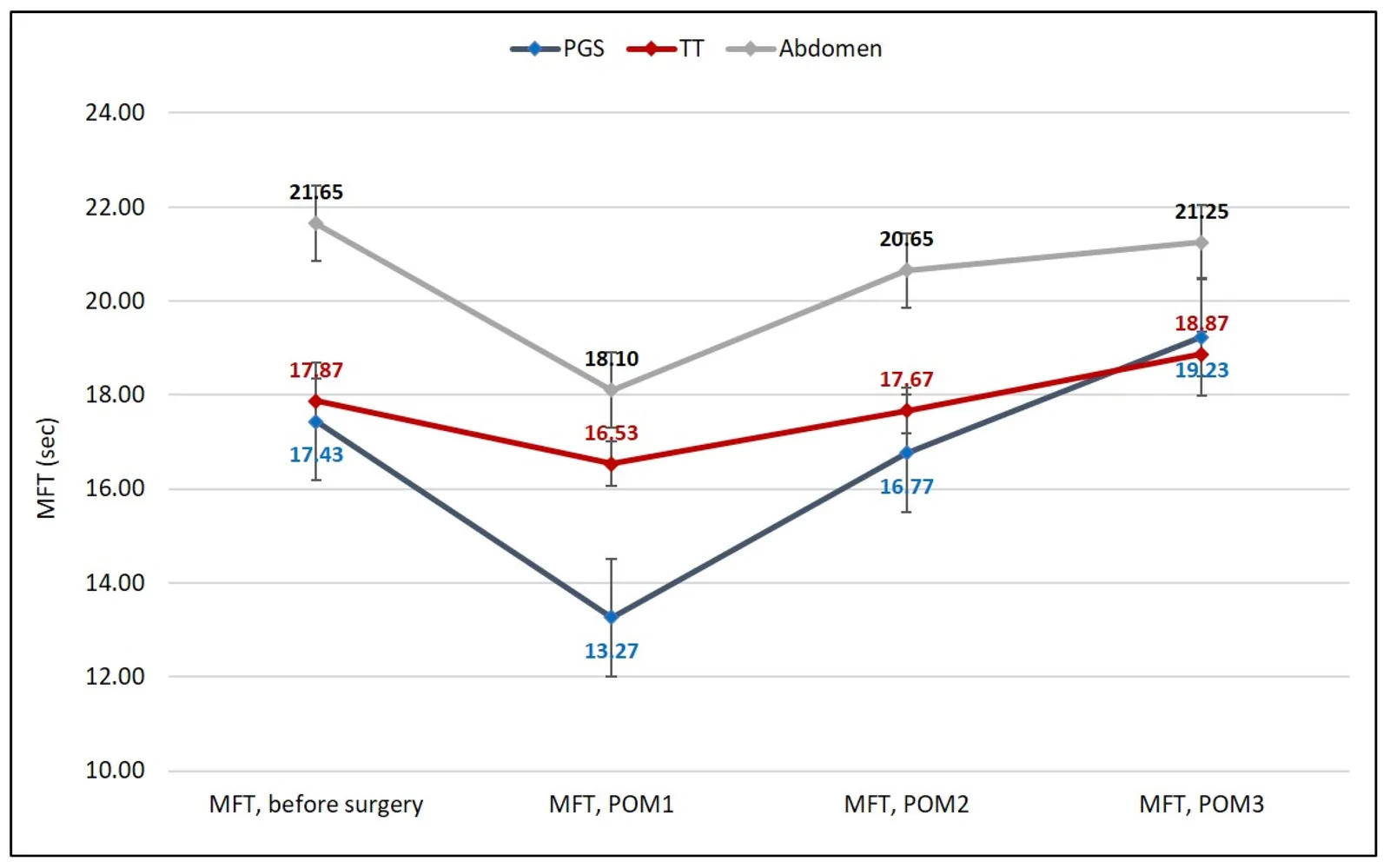
Interaction effect of time and surgery type on MFT. POM—postoperative measurement, MFT—maximum phonation time, PGS—parotid gland surgery, TT—total thyroidectomy, Abdomen—abdominal surgery.

Results show a significant main effect of time on all observed voice parameters and a significant main effect of surgery on all parameters except F_0_. Interaction effects were statistically significant for F_0_ (*F* = 30.511, *p* = 0.000), shimmer (*F* = 10.111, *p* = 0.000), intensity (*F* = 13.873, *p* = 0.000), and MFT (*F* = 6.667, *p* = 0.000), whereas there was no significant interaction between time and type of surgery for jitter. Effect sizes for the main effect of time were larger for shimmer (*ηp*^2^ = 0.338) and smaller for F_0_ (*ηp*^2^ = 0.075), jitter (*ηp*^2^ = 0.161), intensity (*ηp*^2^ = 0.101), and MFT (*ηp*^2^ = 0.068). Effect sizes for the main effect of surgery were relatively small, with the largest for intensity (*ηp*^2^ = 0.253). The effect size of the interaction between time and surgery was highest for F_0_ (*ηp*^2^ = 0.445), somewhat lower for shimmer (*ηp*^2^ = 0.210) and intensity (*ηp*^2^ = 0.267), and lowest for MFT (*ηp*^2^ = 0.149). Means and interaction effects, determined using the Bonferroni post hoc pairwise comparison for each voice parameter, are presented in Figure 2, Figure 3, Figure 4, Figure 5 and Figure 6.

All five observed voice parameters—F_0_, jitter, shimmer, intensity, and MFT—differed significantly across all four measurements (preoperatively, POM1, POM2, and POM3) (Table 3).

At POM1, F_0_ decreased significantly compared with the preoperative measurement; thereafter, we observed a gradual recovery across all three patient groups. A significant difference was found between the group undergoing abdominal surgery and the other two groups (Tukey post hoc comparison). In the preoperative measurement, differences between the groups were smaller. Because normal F_0_ differs between men and women and the groups differ in sex ratio, these differences are probably because head and neck surgeries were mostly performed on women and abdominal surgeries on men. At POM1, abdominal surgeries had the lowest F_0_, and TT the highest. By POM3, the recovery curve shows faster recovery in patients who had PGS or abdominal surgery, while voice changes were more persistent in patients undergoing TT.

At POM1, jitter increased significantly across all three groups and then began to recover by POM2. A significant difference was found between the abdominal surgery and TT groups (Tukey post hoc comparison). In the preoperative measurement, there were no differences between the groups. At POM1, the lowest average jitter was in the abdominal surgery group (M = 2.36), while the highest was observed in the PGS group (M = 2.73). By POM3, recovery had occurred among participants who underwent PGS and abdominal surgery, whereas the mean jitter change in the TT group was somewhat more persistent, though this interaction was not statistically significant.

A significant difference in shimmer was found between the TT group and the other two groups (Tukey post hoc comparison). No differences in shimmer were observed among groups in the preoperative measurement. At POM1, the abdominal surgery group had the lowest mean shimmer (M = 16.18), whereas the PGS group had the highest (M = 18.83). A significant recovery in PGS was observed at POM2, whereas increased shimmer persisted in the TT group, even at POM3.

Importantly, the Tukey post hoc pairwise comparison for shimmer showed that the TT group differed significantly not only from the abdominal surgery group but also directly from the PGS group. Both procedures involve neck manipulation but differ in the extent of laryngeal/tracheal fixation and dissection. This direct TT-versus-PGS contrast supports the conclusion that persistent postoperative shimmer elevation is specifically associated with total thyroidectomy rather than with head and neck surgery in general.

A significant difference in intensity was observed between the abdominal surgery group and the other two groups. Minor differences in preoperative measurements were attributable to differing sex ratios across groups (male and female patients have different ranges of voice intensity). At POM1, the PGS group developed the most severe voice disorder and showed the lowest average intensity (M = 57.27), whereas the abdominal surgery group had the highest average intensity after surgery (M = 68.65). At POM2, the PGS group showed significant recovery, whereas the TT group again exhibited more persistent voice changes. However, by POM3, the TT group’s intensity values also recovered.

At POM1, MFT changed significantly across all three groups, with gradual recovery evident by POM2 and POM3. Significant differences between the abdominal surgery group and the other two groups were detected (Tukey post hoc comparison). As with voice intensity and F_0_, preoperative group differences were attributable to sex ratios. At POM1, PGS had the shortest average phonation time (M = 13.27), whereas abdominal surgery had the longest (M = 18.10). By POM3, all three groups showed significant recovery, but MFT values remained lowest in the TT group.

In the second part of this study, we examined correlations among acquired sociodemographic factors (sex, age, BMI), properties of the surgical and anesthetic procedures (tube size, duration of surgery), and objective voice parameters at each postoperative time point, regardless of surgical type. Pearson correlation coefficients for each postoperative time point—POM1, POM2, and POM3—are presented in Table 4, Table 5 and Table 6.

At POM1, significant negative correlations were observed between the duration of surgery and F_0_ (*r* = −0.244, *p* < 0.05), between the duration of surgery and intensity (*r* = −0.264, *p* < 0.05), and between tube size and F_0_ (*r* = −0.344, *p* < 0.01). A significant positive correlation was observed between tube size and shimmer (*r* = 0.238, *p* < 0.05).

At POM2, a significant positive correlation was observed between surgery duration and shimmer (*r* = 0.252, *p* < 0.05). Significant negative correlations were observed between age and MFT (*r* = −0.282, *p* < 0.05), the duration of surgery and F_0_ (*r* = −0.244, *p* < 0.05), and tube size and shimmer (*r* = −0.238, *p* < 0.05).

At POM3, significant positive correlations were observed between sex and Shimmer (*r* = 0.269, *p* < 0.05), surgery duration and Shimmer (*r* = 0.244, *p* < 0.05), and BMI and Shimmer (*r* = 0.291, *p* < 0.01). Significant negative correlations were observed between sex and intensity (*r* = −0.400, *p* < 0.01), age and MFT (*r* = −0.266, *p* < 0.05), and tube size and Shimmer (*r* = −0.243, *p* < 0.05).

Correlations between sex and F_0_, and between sex and MFT, detected at all three time points are expected because of different normative values for men and women.

These findings indicate that postoperative voice alterations are not only statistically significant but also exhibit distinct recovery patterns by surgical type.

## 4. Discussion

Short-term intubation may lead to clinically relevant voice changes detectable by acoustic analysis, though research on this specific question remains limited [1,2,17]. Additionally, very few studies address the consequences of short-term endotracheal intubation in head and neck surgeries, which pose their own challenges, including specific positioning, frequent head movements, and collisions between the anesthesiologist and the head and neck surgical field during the so-called shared airway.

It should also be emphasized that postoperative voice change after head and neck surgery is unlikely to be attributable to endotracheal intubation alone. In total thyroidectomy, division and retraction of the strap muscles, fixation of the larynx and trachea to surrounding tissue, perilaryngeal/perithyroidal edema, and subclinical dysfunction of the external branch of the superior laryngeal nerve (which is not routinely identified or monitored) are recognized as intubation-independent contributors to early voice change. In parotidectomy, manipulation and retraction near the facial nerve trunk and upper neck, together with local soft-tissue swelling, can similarly affect laryngeal positioning and vocal effort, even though the airway itself is not manipulated. Because our abdominal surgery group underwent anatomically comparable intubation but no neck dissection, the greater and more persistent changes observed in the head and neck groups—particularly after TT—likely reflect a combined effect of intubation plus these surgery-specific mechanical and neural factors, rather than intubation in isolation. This distinction has been made more explicit in the Discussion.

Studies confirm that voice changes occur after thyroidectomy, even without laryngeal nerve injury, which occurs in more than 30% of operated patients. These changes can persist for up to 2 years after surgery, depending on the extent of the procedure and other factors [9,18,19,20,21]. They also affect quality of life [22]. Broader evidence on objective and functional voice outcomes after laryngeal surgery, including reinnervation procedures, similarly underscores that recovery of voice quality after laryngeal-region surgery is often gradual and incompletely captured by short follow-up windows [23].

Derya Abes et al. conducted an acoustic analysis of patients who underwent ear surgery, grouped by surgical duration, and investigated the impact of endotracheal intubation on voice and its duration by performing preoperative and postoperative voice analyses [24]. Patients were divided into three groups based on surgical duration. On the first postoperative day, jitter (%) (*p* = 0.008), shimmer (%) (*p* = 0.027), and shimmer dB (*p* = 0.025) increased significantly with surgical duration, whereas the noise-to-harmonic ratio (NHR) decreased (*p* = 0.028). The authors concluded that endotracheal intubation causes changes in voice in the early postoperative period, but these changes regress over the long term.

We identified a single study comparing two types of head and neck surgery. Sung et al. prospectively analyzed voice outcomes in 155 consecutive patients who underwent thyroidectomy for up to 2 years. The control group consisted of 69 patients who underwent parotidectomy [10]. Acoustic voice analysis was performed using the Multidimensional Voice Program and Voice Range Profile in the Computerized Speech Lab software (Model 4150B, Kay PENTAX/PENTAX Medical, Lincoln Park, NJ, USA) [18]. We analyzed the following parameters: fundamental frequency (F_0_, Hz), jitter (%), shimmer (%), and noise-to-harmonic ratio (NHR, dB). F_0_, jitter, and NHR did not change significantly after surgery in either the cases or the control groups, except at occasional time points. Shimmer was significantly worse for 12 months after thyroidectomy, except in the first week postoperatively. F_0_, jitter, shimmer, and NHR did not differ significantly between the case and control groups at most time points. Self-assessed voice symptoms and effects on objective acoustic parameters persisted for up to 18 months after thyroidectomy. This possibility should be explained to patients and discussed during preoperative consultations.

In this study, we compared short-term voice recovery patterns after head–neck and abdominal surgeries. All patient groups showed statistically significant voice changes immediately after surgery, but the head–neck group had the largest and longest-lasting changes—especially among those undergoing TT. This pattern was observed across most acoustic measures (F_0_, jitter, shimmer, intensity, MFT), indicating a robust effect of TT and intubation on voice. Although the abdominal surgery group also showed transient changes (likely due to intubation alone), these resolved more quickly. Thus, the data suggest that both intubation and proximity of surgery to laryngeal structures contribute to short-term voice disturbance.

Shimmer showed the largest overall effect size among the parameters studied, which is physiologically plausible. Shimmer reflects short-term, cycle-to-cycle amplitude perturbations of vocal fold vibration and is particularly sensitive to incomplete or asymmetric glottal closure, mucosal edema, and irregular vocal fold mass or stiffness—changes readily produced by intubation-related mucosal trauma and by surgery-related edema or altered laryngeal positioning. Jitter, by contrast, mainly reflects cycle-to-cycle frequency instability governed by neuromuscular control of fold tension and is comparatively more robust to mild mucosal or biomechanical disturbance. F_0_ depends heavily on vocal fold tension and length and is strongly influenced by sex, which may dilute its sensitivity as a group-level marker despite being included as a covariate. This pattern is consistent with previous reports identifying shimmer as one of the most sensitive indices of early, subclinical laryngeal dysfunction after intubation and neck surgery [10,24].

At POM1, surgery duration and tube size were important predictors of voice disorder intensity for some voice parameters. Longer surgeries were associated with lower F_0_ and lower voice intensity, while larger tube size was associated with lower F_0_ and higher shimmer. Previous studies also confirm that longer exposure to intubation and inadequate tube size can cause more injuries and, as a result, more severe voice disorders [1,3,25]. However, an interesting finding is that the correlation between potential risk factors and voice parameters changes over time. At POM2, older participants had shorter phonation times, suggesting that their phonation capability was recovering more slowly than in younger patients. Finally, at POM3, women had higher shimmer scores. Given that most TT patients were women, these results confirm earlier findings that the TT group is recovering more slowly than the other two groups. Additionally, longer surgery duration was associated with higher shimmer, while larger tube size was associated with lower shimmer. These results likely reflect that men, on average, have larger tubes and are predominantly in the abdominal surgery group, which recovered faster. So again, these results point to patients undergoing TT recovering the slowest.

One month after surgery, BMI was among the most important indicators of persistent voice disorder. In our previous study, we investigated the prevalence and predictors of voice disorders among thyroidectomy patients without recurrent laryngeal nerve injury [26]. The study analyzed 243 participants. Significant associations were observed for surgery type (*χ*^2^ = 29.88, *p* < 0.001), with total thyroidectomy carrying a higher risk; surgery duration (*χ*^2^ = 16.40, *p* < 0.001); thyroid volume (*χ*^2^ = 4.24, *p* = 0.045); and BMI (*χ*^2^ = 8.97, *p* = 0.011). Multivariate analysis confirmed sex and surgery duration as significant contributors. Significant acoustic differences were also linked to BMI categories, with obese participants exhibiting poorer parameters, particularly shimmer and jitter. In this study, higher BMI was associated with higher shimmer one month after surgery. Given the lack of significant differences in BMI among the three patient groups, these results indicate that participants with higher BMI recover more slowly.

This correlation, however, was modest in magnitude (*r* = 0.291) and should be interpreted as a weak association rather than as evidence of a causal or strongly predictive relationship. BMI alone explains only a small proportion of the variance in POM3 shimmer, and unmeasured factors likely contribute substantially more to persistent voice change.

Mechanistically, higher BMI may prolong voice recovery by reducing pulmonary reserve and altering respiratory support for phonation, increasing perilaryngeal and neck soft-tissue mass that slows resolution of postoperative edema, and increasing the prevalence of subclinical laryngopharyngeal reflux, all of which can compound intubation-related mucosal injury. The sex-related shimmer difference observed at POM3 is most parsimoniously explained by the disproportionate representation of women in the TT group (the slowest-recovering group) rather than by an independent biological effect of sex on shimmer recovery, since sex was already included as a covariate; still, baseline sex differences in laryngeal anatomy and vocal fold mass may also modulate the biomechanical response to surgical trauma. Because surgery duration, tube size, and BMI were examined through separate bivariate correlations rather than a single multivariable model, we cannot rule out collinearity among these perioperative variables or determine which represents the strongest independent predictor of voice outcome; a multivariable regression adjusting simultaneously for surgery type, duration, tube size, and BMI would be needed to establish independent risk factors, and we identify this as a priority for a follow-up analysis of this cohort.

Our findings confirm that endotracheal intubation is associated with short-term postoperative voice changes, regardless of the type of surgery. However, the intensity and duration of these changes vary by procedure.

Patients undergoing abdominal surgery had milder, shorter-lasting voice changes, whereas those undergoing head and neck procedures had more pronounced changes. Among these, patients undergoing total thyroidectomy had the slowest recovery, with some parameters remaining outside normal ranges even one month postoperatively. These findings suggest that, in addition to intubation, surgical factors such as anatomical proximity to the larynx, intraoperative manipulation, and positioning may contribute to postoperative voice outcomes. Further research is needed to clarify the mechanisms and long-term implications of voice changes after short-term endotracheal intubation. Although the observed changes in acoustic voice parameters were statistically significant, their clinical relevance should be interpreted cautiously. In most cases, these changes are likely to be mild and transient. However, they may still be noticeable to patients, particularly in the early postoperative period. Such changes may be important for individuals who rely heavily on their voice professionally—such as teachers, singers, broadcasters, call-center operators, and other professional voice users—for whom even mild, transient postoperative dysphonia may temporarily impair work performance, warrant closer preoperative counseling and postoperative voice rest or guidance, and could affect everyday functions such as communication or the use of voice recognition technologies. Therefore, clinicians should not underestimate even short-term voice alterations.

Several study limitations should be recognized. First, no formal sample size calculation was conducted, which may have affected statistical power; the abdominal surgery group was also the smallest (*n* = 20 vs. *n* = 30 in each head/neck group), and, together with the significantly unequal sex distribution across groups (Figure 1), this raises the possibility of statistical bias, particularly for comparisons involving the abdominal group.

Sex was therefore retained as a covariate in all ANCOVA models to reduce, though not fully eliminate, the influence of this imbalance; nonetheless, replication in a larger, prospectively powered, and more sex-balanced cohort is warranted before generalizing these findings.

Second, variations in sex distribution and tube size between groups could limit direct comparisons, although sex was accounted for as a covariate in the analysis.

Third, because the study is observational, we cannot establish causality, and residual confounding may still exist.

Fourth, follow-up was limited to POM3 (one month postoperatively). This endpoint was chosen because it captures the early, clinically most relevant recovery window, during which most transient intubation-related voice changes resolve, and patients typically return to work or usual activities. It also matched the follow-up horizon of the previously published study protocol on which the acoustic assessment method was based. However, thyroidectomy-related voice recovery is known to extend over several months, and some patients in the TT group still had abnormal shimmer and jitter values at POM3 (see Section 3). The one-month endpoint therefore likely underestimates the true duration of postoperative voice impairment, particularly after TT. Our conclusions about “persistent” change should be understood as ‘’persistent within the first postoperative month’’ rather than as reflecting long-term outcome. Longer follow-up (e.g., 3–6 months) is needed to determine when, or whether, complete recovery occurs.

Furthermore, retrospective data collection meant that detailed information on intubation techniques, the number of attempts, cuff pressure, and other anesthesia-related factors was not consistently available.

Number of intubation attempts, Cormack-Lehane grade, cuff pressure, and use of a stylet are well-established determinants of postoperative laryngeal morbidity, and their absence here is a substantive limitation, not merely a methodological detail: unmeasured differences in intubation difficulty or technique between surgical groups could partially confound the observed associations between surgery type and voice outcomes, and this possibility cannot be ruled out with the present data.

These variables are known to affect laryngeal injury and postoperative voice outcomes. Additionally, although objective acoustic measures were used, the study did not include subjective patient reports or clinical voice assessments, potentially limiting insights into real-world effects.

In particular, the study did not use a validated patient-reported voice handicap instrument (e.g., Voice Handicap Index) or obtain postoperative laryngoscopic/stroboscopic grading. Subclinical acoustic changes, such as those reported here, do not necessarily correspond to a patient-perceived voice handicap or to a visible laryngeal finding, so the real-world symptomatic and functional burden of these changes remains to be established in future studies combining acoustic, patient-reported, and laryngoscopic assessment.

## 5. Conclusions

Short-duration endotracheal intubation is associated with significant, measurable deterioration in objective acoustic voice parameters, particularly in patients undergoing head and neck surgery. Shimmer appears to be the most sensitive indicator of postoperative voice impairment. These findings support early postoperative acoustic voice assessment to detect subclinical laryngeal dysfunction and underscore the importance of appropriate preoperative patient counseling and careful airway management. Further controlled studies are needed to clarify the underlying mechanisms and to optimize prevention and treatment strategies.

## Figures and Tables

**Table 1:** Baseline characteristics of study participants.

		N	M	SD	Min	Max	F	Sig.
**Age**	PGS	30	51.8667	14.25079	27.00	86.00	1.673	0.195
	TT	30	53.0667	12.62983	29.00	78.00		
	Abdomen	20	46.2500	13.23025	18.00	65.00		
	Total	80	50.9125	13.52091	18.00	86.00		
**BMI**	PGS	30	25.9770	3.46541	16.98	32.49	2.255	0.112
	TT	30	27.3800	4.83232	19.81	36.33		
	Abdomen	20	25.1000	2.47833	21.20	31.00		
	Total	80	26.2839	3.91209	16.98	36.33		
**Duration of surgery**	PGS	30	125.6667	46.54870	45.00	225.00	21.491	0.000
	TT	30	126.5000	29.68658	70.00	180.00		
	Abdomen	20	65.4000	23.59839	25.00	135.00		
	Total	80	110.9125	44.18351	25.00	225.00		
**Tube size**	PGS	30	7.3833	0.48572	6.00	8.00	7.222	0.001
	TT	30	7.0833	0.34947	6.50	7.50		
	Abdomen	20	7.5000	0.36274	7.00	8.00		
	Total	80	7.3000	0.44008	6.00	8.00		

BMI—body mass index, TT—total thyroidectomy, PGS—parotidectomy.

**Table 2:** Descriptive statistics for observed voice parameters.

	N	Min	Max	M	SD	Skewness	Kurtosis
**F_0_**	80	120.00	216.00	165.41	30.614	−0.348	−1.536
POM1	80	52.00	200.00	131.13	37.809	−0.246	−1.406
POM2	80	89.00	220.00	145.16	32.888	−0.266	−1.251
POM3	80	98.00	219.00	156.58	29.854	−0.244	−1.250
**Jitter**	80	0.01	0.50	0.27	0.139	0.129	−1.164
POM1	80	0.75	6.98	2.60	1.171	1.104	1.935
POM2	80	0.11	3.56	0.90	0.590	1.794	4.760
POM3	80	0.01	2.45	0.53	0.424	1.518	3.920
**Shimmer**	80	1.20	6.15	3.68	1.069	−0.521	0.074
POM1	80	8.12	35.88	17.18	5.706	0.858	1.044
POM2	80	4.12	19.25	10.53	3.376	0.494	−0.210
POM3	80	1.12	16.21	6.59	3.936	0.930	−0.268
**Intensity**	80	62.00	75.00	69.83	2.666	−0.165	0.144
POM1	80	30.00	79.00	62.98	8.083	−1.088	2.780
POM2	80	60.00	78.00	66.99	4.002	0.340	−0.289
POM3	80	60.00	77.00	68.50	3.257	0.091	0.033
**MFT**	80	10.00	25.00	18.65	3.569	0.081	−0.785
POM1	80	7.00	25.00	15.70	4.724	−0.139	−1.042
POM2	80	9.00	25.00	18.08	3.778	0.018	−0.924
POM3	80	11.00	28.00	19.60	3.255	0.074	−0.228

POM—postoperative measurement, F_0_—fundamental frequency (voice pitch), MFT—maximum phonation time.

**Table 3:** Main effects of time and type of surgery, and their interaction, on objective voice parameters.

	Main Effect of Time				Main Effect of Surgery				Interaction			
	*F*	*df*	*p*	*ηp* ^2^	*F*	*df*	*p*	*ηp* ^2^	*F*	*df*	*p*	*ηp* ^2^
**F_0_**	6.193	2	0.002	0.075	0.768	2	0.468	0.020	30.511	4	0.000	0.445
**Jitter**	14.631	1	0.000	0.161	3.495	2	0.035	0.084	2.356	3	0.076	0.058
**Shimmer**	38.813	2	0.000	0.338	8.517	2	0.000	0.183	10.111	4	0.000	0.210
**Intensity**	8.547	2	0.001	0.101	12.881	2	0.000	0.253	13.873	3	0.000	0.267
**MFT**	5.521	2	0.004	0.068	5.252	2	0.007	0.121	6.677	4	0.000	0.149

F_0_—fundamental frequency (voice pitch), MFT—maximum phonation time.

**Table 4:** Correlation matrix for POM1.

	Sex	Age	Duration	Tube	BMI	F_0_	Jitter	Shimmer	Intensity
**Age**	0.046								
**Duration**	0.135	0.211							
**Tube**	−0.422 **	−0.021	0.076						
**BMI**	−0.018	0.281 *	0.136	0.046					
**F_0_**	0.748 **	0.059	−0.244 *	−0.344 **	0.079				
**Jitter**	0.075	0.160	−0.006	0.075	0.116	0.053			
**Shimmer**	−0.203	0.151	0.215	0.238 *	0.052	−0.115	−0.043		
**Intensity**	−0.031	0.024	−0.264 *	−0.037	−0.050	−0.081	−0.122	−0.072	
**MFT**	−0.237 *	−0.176	0.004	0.016	0.118	−0.273 *	−0.090	−0.161	0.120

**. Correlation is significant at the 0.01 level (2-tailed). *. Correlation is significant at the 0.05 level (2-tailed). BMI—body mass index, F_0_—fundamental frequency (voice pitch), POM—postoperative measurement.

**Table 5:** Correlation matrix for POM2.

	Sex	Age	Duration	Tube	BMI	F_0_	Jitter	Shimmer	Intensity
**Age**	0.046								
**Duration**	0.135	0.211							
**Tube**	−0.422 **	−0.021	0.076						
**BMI**	−0.018	0.281 *	0.136	0.046					
**F_0_**	0.829 **	0.000	0.024	−0.301 **	−0.025				
**Jitter**	0.065	0.162	0.041	−0.061	0.217	0.101			
**Shimmer**	0.143	0.071	0.252 *	0.125	0.159	0.182	0.192		
**Intensity**	−0.086	−0.185	−0.127	0.146	−0.165	0.009	−0.271 *	−0.316 **	
**MFT**	−0.415 **	−0.282 *	−0.095	0.158	0.029	−0.334 **	−0.136	0.033	0.049

**. Correlation is significant at the 0.01 level (2-tailed). *. Correlation is significant at the 0.05 level (2-tailed). BMI—body mass index, F_0_—fundamental frequency (voice pitch), POM—postoperative measurement.

**Table 6:** Correlation matrix for POM3.

	Sex	Age	Duration	Tube	BMI	F_0_	Jitter	Shimmer	Intensity
**Age**	0.046								
**Duration**	0.135	0.211							
**Tube**	−0.422 **	−0.021	0.076						
**BMI**	−0.018	0.281 *	0.136	0.046					
**F_0_**	0.775 **	−0.049	−0.138	−0.185	−0.041				
**Jitter**	0.182	0.128	0.145	−0.198	0.196	0.021			
**Shimmer**	0.269 *	0.170	0.244 *	−0.243 *	0.291 **	0.090	0.687 **		
**Intensity**	−0.400 **	−0.141	0.083	0.079	−0.012	−0.327 **	−0.181	−0.063	
**MFT**	−0.408 **	−0.266 *	−0.007	0.191	0.045	−0.342 **	−0.181	−0.085	0.278 *

**. Correlation is significant at the 0.01 level (2-tailed). *. Correlation is significant at the 0.05 level (2-tailed). BMI—body mass index, F_0_—fundamental frequency (voice pitch), POM—postoperative measurement.

## Data Availability

The original contributions presented in this study are included in the article. Further inquiries can be directed to the corresponding author(s).

## Abbreviations

The following abbreviations are used in this manuscript:

BMI	Body Mass Index
dB	Decibels
ET	Endotracheal Intubation
F_0_	Fundamental Frequency
Hz	Hertz
MFT	Maximum Phonation Time
NHR	Noise-to-Harmonic Ratio
PGS	Parotidectomy
POM	Postoperative Measurement
TT	Total Thyroidectomy

## References

[1] Mencke T., Echternach M., Kleinschmidt S., Lux P., Barth V., Plinkert P.K., Fuchs-Buder T. (2003). Laryngeal morbidity and quality of tracheal intubation: A randomized controlled trial. Anesthesiology.

[2] Yamanaka H., Hayashi Y., Watanabe Y., Uematu H., Mashimo T. (2009). Prolonged hoarseness and arytenoid cartilage dislocation after tracheal intubation. Br. J. Anaesth..

[3] Shinn J.R., Kimura K.S., Campbell B.R., Sun Lowery A., Wootten C.T., Garrett C.G., Francis D.O., Hillel A.T., Du L., Casey J.D. (2019). Incidence and outcomes of acute laryngeal injury after prolonged mechanical ventilation. Crit. Care Med..

[4] Kitahara S., Masuda Y., Kitagawa Y. (2005). Vocal fold injury following endotracheal intubation. J. Laryngol. Otol..

[5] Domino K.B., Posner K.L., Caplan R.A., Cheney F.W. (1999). Airway injury during anesthesia: A closed claims analysis. Anesthesiology.

[6] Donati F., Plaud B. (2008). Tracheal intubation: Optimal conditions, vocal cord damage, and allergy. Can. J. Anaesth..

[7] Wallace S., McGrath B.A. (2021). Laryngeal complications after tracheal intubation and tracheostomy. BJA Educ..

[8] Šimić I., Curić Radivojević R., Slipac J., Prgomet D. (2023). Voice condition following short-term endotracheal intubation in head and neck surgery: Study protocol for clinical trial. Acta Clin. Croat..

[9] Vicente D.A., Solomon N.P., Avital I., Henry L.R., Howard R.S., Helou L.B., Coppit G.L., Shriver C.D., Buckenmaier C.C., Libutti S.K. (2014). Voice outcomes after total thyroidectomy, partial thyroidectomy, or non-neck surgery using a prospective multifactorial assessment. J. Am. Coll. Surg..

[10] Sung E.S., Kim K.Y., Yun B.R., Song C.M., Ji Y.B., Lee J.C., Tae K. (2018). Long-term functional voice outcomes after thyroidectomy, and effect of endotracheal intubation on voice. Eur. Arch. Otorhinolaryngol..

[11] Brodsky M.B., Levy M.J., Jedlanek E., Pandian V., Blackford B., Price C., Cole G., Hillel A.T., Best S.R., Akst L.M. (2018). Laryngeal injury and upper airway symptoms after oral endotracheal intubation with mechanical ventilation during critical care: A systematic review. Crit. Care Med..

[12] Colton House J., Noordzij J.P., Murgia B., Langmore S. (2011). Laryngeal injury from prolonged intubation: A prospective analysis of contributing factors. Laryngoscope.

[13] Solomon N.P., Helou L.B., Dietrich-Burns K., Stojadinovic A. (2011). Do obesity and weight loss affect vocal function?. Semin. Speech Lang..

[14] IBM Corp IBM SPSS Statistics for Windows, Version 28.0; Released 2021.

[15] Filion G.J. (2015). The signed Kolmogorov-Smirnov test: Why it should not be used. Gigascience.

[16] Steinskog D.J., Tjøstheim D.B., Kvamstø N.G. (2007). A cautionary note on the use of the Kolmogorov–Smirnov test for normality. Mon. Weather Rev..

[17] Mendels E.J., Brunings J.W., Hamaekers A.E., Stokroos R.J., Kremer B., Baijens L.W. (2012). Adverse laryngeal effects following short-term general anesthesia: A systematic review. Arch. Otolaryngol. Head Neck Surg..

[18] Stojadinovic A., Shaha A.R., Orlikoff R.F., Nissan A., Kornak M.F., Singh B., Boyle J.O., Shah J.P., Brennan M.F., Kraus D.H. (2002). Prospective functional voice assessment in patients undergoing thyroid surgery. Ann. Surg..

[19] Beka E., Gimm O. (2024). Voice changes without laryngeal nerve alterations after thyroidectomy: The need for prospective trials—A review study. J. Voice.

[20] Kim G.J., Bang J., Shin H.I., Kim S.Y., Bae J.S., Kim K., Kim J.S., Hwang Y.S., Shim M.R., Sun D.I. (2023). Persistent subjective voice symptoms for two years after thyroidectomy. Am. J. Otolaryngol..

[21] Li C., Lopez B., Fligor S., Broekhuis J.M., Maeda A., Duncan S., Chen H.W., Choudhary A., Budwani S., Hasselgren P.O. (2021). Long-term voice changes after thyroidectomy: Results from a validated survey. Surgery.

[22] D’haeseleer E., Huvenne W., Vermeersch H., Meerschman I., Imke K., Servayge L., Versavel O., Van Lierde K. (2021). Long-term voice quality outcome after thyroidectomy without laryngeal nerve injury: A prospective 10 year follow up study. J. Commun. Disord..

[23] Guarino P., Russo G., Chiari F., Barillari M.R., Camesasca V., Carobbio A.L.C., Filauro M., Iandelli A., Laura E., Lechien J.R. (2025). Laryngeal reinnervation techniques for unilateral vocal fold paralysis—Clinical outcomes and surgical approaches: A systematic review and meta-analysis. J. Voice.

[24] Abeş D., Tüzün K., Tuhanioğlu B., Erkan S.O., Özdaş T., Senkal O.A. (2023). Effect of endotracheal intubation on voice. J. Voice.

[25] Karmali S., Rose P. (2020). Tracheal tube size in adults undergoing elective surgery—A narrative review. Anaesthesia.

[26] Šimić Prgomet I., Frkanec S., Gugić Radojković I., Prgomet D. (2024). Prospective voice assessment after thyroidectomy without recurrent laryngeal nerve injury. Diagnostics.

